# Left Atrial Appendage Closure: A Current Overview Focused on Technical Aspects and Different Approaches

**DOI:** 10.31083/j.rcm2305155

**Published:** 2022-04-26

**Authors:** Fabrizio Guarracini, Marta Martin, Massimiliano Marini, Stefano Branzoli, Giulia Casagranda, Daniele Muser, Giovanni B. Forleo, Alessio Gasperetti, Massimo Di Marco, Stefano Guarracini, Roberto Bonmassari, Patrizio Mazzone, Antonio M Calafiore, Michele Di Mauro

**Affiliations:** ^1^Department of Cardiology, Santa Chiara Hospital, 38122 Trento, Italy; ^2^Department of Cardiac Surgery, Santa Chiara Hospital, 38122 Trento, Italy; ^3^Department of Cardiac Surgery, UZ Brussel, 1090 Brussels, Belgium; ^4^Department of Diagnostic Imaging, APSS of Trento, 37122 Trento, Italy; ^5^Cardiac Electrophysiology, Cardiovascular Medicine Division, Hospital of the University of Pennsylvania, Philadelphia, PA 19104, USA; ^6^Cardiothoracic Department, Udine General Hospital, 33100 Udine, Italy; ^7^Cardiology Unit, ASST-Fatebenefratelli Sacco, Luigi Sacco University Hospital, 20157 Milan, Italy; ^8^Division of Cardiology, Department of Medicine, Johns Hopkins University, Johns Hopkins Hospital, Baltimore, MD 21287, USA; ^9^Department of Cardiology, “Santo Spirito" Hospital, 00193 Pescara, Italy; ^10^Department of Cardiology, “Pierangeli" Hospital, 00193 Pescara, Italy; ^11^Arrhythmology and Cardiac Pacing Unit, San Raffaele Hospital, 20132 Milan, Italy; ^12^Division of Cardiac Surgery A, Henry Dunant Hospital, 115 26 Athens, Greece; ^13^Cardio-Thoracic Surgery Unit, Heart and Vascular Centre, Maastricht University Medical Centre (MUMC), Cardiovascular Research Institute Maastricht (CARIM), 6202 AZ Maastricht, The Netherlands

**Keywords:** left atrial appendage occlusion, atrial fibrillation, bleeding risk, surgical left atrial appendage exclusion

## Abstract

Several studies in literature have shown that 90% of emboli related to non-valvular atrial fibrillation originate from left atrial appendage. Percutaneous closure or surgical exclusion of left atrial appendage in patients with high bleeding and high cardioembolic risk is currently a well established procedure in literature, clinical practice and guidelines. Knowledge of different techniques of left atrial appendage closure is necessary to individualize the procedure according to the patient anatomy and pre-procedural imaging evaluations. In this review the authors will evaluate different left atrial appendage closure systems and the different pre and intra procedural imaging methods.

## 1. Introduction

Left atrial appendage occlusion (LAAO) is a technique used since 2001 to reduce the risk of ischemic stroke in patients with non-valvular atrial fibrillation (AF) and contraindication to long-term anticoagulation therapy (OAT) [1].

This procedure develops from evidence that AF determines approximately 15–20% of ischemic strokes and in more than 90% of cases the source of thrombotic formations is located in the left atrial appendage (LAA) [1].

Recent guidelines and previous studies in literature indicate the use of long term OAT in high risk patients based on the ${\mathrm{CHA}}_{2}$${\mathrm{DS}}_{2}$–VASc score even if underwent to interventional procedures (transcatheter or surgical AF ablation) [2, 3].

Although warfarin is highly effective in the prevention of stroke and systemic embolism, its use is limited by a narrow therapeutic range, numerous food and drug interactions, and an increased risk of bleeding [4]. In addition, many factors influence the intensity of the anticoagulation effect. The most common were used to validate a score, ${\mathrm{SAMe-TT}}_{2}$$R_{2}$, identifying patients who are less likely to maintain an adequate therapeutic range [3].

The percentage of patients who are unable to maintain an adequate therapeutic range is around 30–40%. Furthermore, due to complex therapy management, 30% of patients discontinue OAT within 1 year [5].

Most of these disadvantages have been partially mitigated by new oral anticoagulants (NOAC), which, in the face of a 51% reduction in risk of hemorrhagic stroke, however, result in a significant increase in the risk of gastrointestinal bleeding, compared to warfarin, and cannot be taken in case of severe nephropathy or liver disease [6]. In any case, taking NOAC results in an increased risk of bleeding, compared to the absence of any antithrombotic therapy.

For stratification of bleeding risk, the literature data agrees with the use of the HAS-BLED score, which identifies high risk patients with ≥3 points in the score. This value does not contraindicate OAT but requires closer follow-up and elimination of correctable hemorrhagic risk factors [3].

On the basis of these issues related to OAT and following the results of the already widespread surgical exclusion of LAA, creation of different devices for percutaneous approach were developed.

## 2. Evidences in Literature and Guidelines Indications

There are currently only three randomized trials of percutaneous LAAO comparing device versus OAT.

PROTECT-AF study [7] (Watchman Left Atrial Appendage System for Embolic PROTECTion in Patients With Atrial Fibrillation) is a multicenter, randomized, prospective, non-inferiority study that randomized 707 patients with non-valvular AF and a theoretical indication for OAT (warfarin), percutaneous LAAO with the Watchman device (and OAT therapy for 45 days post-procedure) versus long term OAT.

Watchman showed to be non-inferior to OAT (18 month follow-up) in preventing the composite endpoint of death, ischemic stroke, hemorrhagic stroke, and peripheral embolic events.

Although there were significantly more adverse events in patients treated invasively than in the control group, there was a progressive reduction in the incidence of learning-related complications during the study. This reduction was confirmed by the subsequent Continued Access Protocol (CAP) registry [8].

PREVAIL study [9] enrolled 407 patients who were randomized to percutaneous LAAO or long therm warfarin therapy. The interventional arm has been shown to be non-inferior to OAT in preventing ischemic stroke or peripheral emboli. The results of this study also showed low complication rates (major device or procedure-related complications decreased from 8.7% in the PROTECT-AF study to 4.4% in the current study), for first-time implanters and experienced clinicians both. However, for patients not eligible for long therm OAT therapy, data is limited.

Recently PRAGUE– 17 trial, a randomized trial with 4 years follow up and 402 high risk patients with AF (${\mathrm{CHA}}_{2}$${\mathrm{DS}}_{2}$-VASc 4.7+1.5, HASBLED 3.1+0.9), demonstrated percutaneous LAAO was non inferior to NOAC for reducing major cardiovascular, neurological or bleeding events in high risk patients with AF. In the study the authors also found that non-procedural bleeding was significantly reduced with percutaneous LAAO [10].

Therefore ASAP study [11] (ASA Plavix Feasibility Study With Watchman Left Atrial Appendage Closure Technology) is a non-randomized feasibility study designed to establish safety and effectiveness of Watchman device in patients with AF non eligible to warfarin therapy. 150 patients with AF treated with dual antiplatelet therapy (ASA and Plavix) were evaluated for six months post-procedure. Procedure- or device-related safety events occurred in 8.7% of patients. Patients were followed for an average follow-up of 14.4 months. In particular in this population data demonstrated a 77% reduction in risk of ischemic stroke (1.7% annual ischemic brain events vs expected 7.3% based on the ${\mathrm{CHADS}}_{2}$ scores of the patient cohort) and a significant reduction of hemorrhagic stroke expected.

Despite these results, in the 2020 European Society of Cardiology Guidelines for the diagnosis and management of atrial fibrillation developed in collaboration with the European Association for Cardio-Thoracic Surgery (EACTS) guidelines, percutaneous LAAO remains in class IIb (level of evidence B) in patients with AF and long-term OAT contraindication for stroke prevention [3].

Indeed, as reported also in 2019 EHRA/EAPCI consensus, patients with high risk of bleeding due to characteristics not considered by the HAS-BLED score, patients with cardio-embolic events despite OAT therapy, patients with electrically isolated left atrial appendage post catheter ablation and non-compliant patients, patients with end-stage renal disease could be considered for LAAO [12].

## 3. Periprocedural and Intraprocedural Imaging Assessment 

The use of different imaging techniques, including integrated imaging evaluation, is critical for selection of patient, device, procedure monitoring and subsequent follow-up.

### 3.1 Transthoracic Echocardiogram

This non-invasive method is critical in both initial patient assessment and follow-up. It is also essential for identify any contraindications to the procedure, such as severe mitral stenosis and/or the presence of ventricular thrombosis. It is recommended for all patients to evaluate cardiac function (ejection fraction), left atrial and mitral valve apparatus dimensions. After the procedure, it is useful to rule out the presence of pericardial effusion or device embolization.

### 3.2 Transesophageal Echocardiography

Transesophageal Echocardiography (TEE) is currently considered principal method for excluding LAA thrombus, for the anatomical evaluation with cardiac computed tomography (CT), for morphological analysis of LAA and selection of device size for closure. It is also the most common method for intraprocedural imaging.

TEE can detect thrombus in LAA with high sensitivity (92%) and specificity (98%) also in complex anatomy. Even if elements may overestimate the diagnosis of LAA thrombus (pectinate muscles, prosthetic valve artifacts etc.) the positive predictive value of this method remains high (86%). In addition, the use of a contrast medium may further aid in this assessment [12]. This two-dimensional method and more the development of 3D ultrasound TTE is critical to the evaluation of LAA morphology (cactus, chicken wing, windsock type etc.) and its size [13]. A first assessment should be performed along the horizontal short axis at the base of the heart and in two chambers (longitudinal). The multiplanar method allows for a complete visualization with intermediate planes.

3D evaluation, in addition to being more closely correlated with the cardiac CT, allows a more accurate definition of the morphology of LAA and its measurements, the volume and ejection fraction calculation derived from the volume [14]. In addition, such reconstruction, compared to 2D, is more effective in assessing the structure (calcifications) and the mobility of the thrombus itself.

A useful tool for LAA assessment is pulsed Doppler sonography for the detection of maximum flow rate (usually measured at the proximal third of the LAA): when this rate is found to be greater than 40 cm/sec it correlates with a low risk of thrombotic formations; below this value there is a higher response of spontaneous echo-contrast (SEC) and stroke risk. Values less than 20 cm/sec correlate with the presence of LAA thrombus and increased incidence of thromboembolic events. The presence of SEC and slow flow within the LAA hinders the safe exclusion of thrombi in the left atrial appendage. Assessment of diastolic function is also helpful as high filling pressure can contribute to stasis and thus thrombus formation in LAA [15].

The use of ultrasound contrast agent composed of microbubbles demonstrated to improve the diagnostic performance of TEE. The main contrast mechanism is based on the difference in density and compressibility between microbubbles and the surrounding environment, thus creating an efficient ultrasound reflector and improved blood echogenicity [16].

**Fig. 1.**
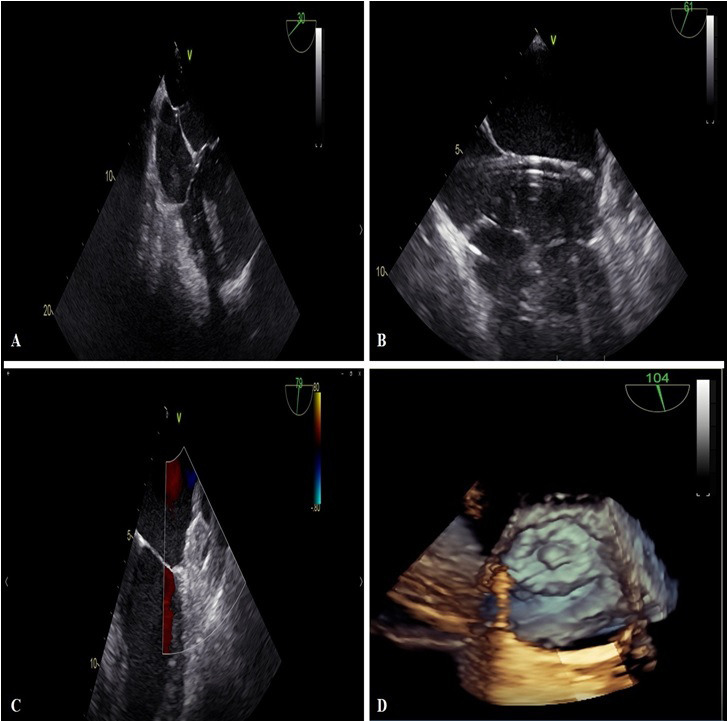
**TEE monitoring during percutaneous LAAO with Watchman Flex device**. (A) Highlights the transseptal puncture maneuver. (B) Describe device positioning with double curve sheath. (C,D) Highlights in 2 D and 3 D TEE modality the complete occlusion of LAA.

During percutaneous LAAO and TEE monitoring it is necessary that an expert operator accurately identifies the projections useful for a generic re-evaluation at the beginning of the procedure, for the transseptal puncture, for the correct sizing and for the correct positioning of the device (Fig. 1).

First of all, it is necessary to obtain the best visualization of LAA along the long axis that is normally achieved with the probe placed at medium level in the esophagus with a plane between ${\mathrm{50}}^{\circ }$ and ${\mathrm{70}}^{\circ }$. The degrees vary depending on the location of LAA (e.g., with a more anterior LAA it will be necessary to move between $0^{\circ }$ and ${\mathrm{50}}^{\circ }$; with a more lateral LAA between ${\mathrm{70}}^{\circ }$ and ${\mathrm{90}}^{\circ }$). The best projection is in a ${\mathrm{135}}^{\circ }$ plane to visualize the anterior and posterior portion of LAA along the short axis. This projection is also the best one to decide the size of the device as in most cases it shows the major axis of LAA.

Each device needs different sizing measurements that should be evaluated in 4 different projections ($0^{\circ }$, ${\mathrm{45}}^{\circ }$, ${\mathrm{90}}^{\circ }$ and ${\mathrm{135}}^{\circ }$) to identify the largest size.

For the use of the Watchman (Boston Scientific, Natick, MA, USA) device is necessary evaluate a line from the circumflex coronary artery to a point 1–2 cm within the ridge end of the superior pulmonary vein (anatomical orifice) and it is essential also measure the depth of LAA.

For the use of Amplatzer Amulet device (St. Jude Medical, St. Paul, Minnesota, USA) is crucial measure 12 mm from the line connecting the circumflex coronary artery to the pulmonary vein (landing zone). In this case the depth of LAA is less significant.

Therefore TEE helps the operator perform a safe transseptal puncture. LAA is often oriented anteriorly and laterally, a posterior and slightly inferior transseptal puncture allows for proper alignment of the guide catheter with the major axis of the atrial appendage.

To allow safe performance of this procedure it is often necessary to start with a projection at 90–${\mathrm{100}}^{\circ }$ (cranio-caudal) and to then switch to ${\mathrm{45}}^{\circ }$ (short axis) once the needle has approached the fossa ovalis.

The interatrial septum thickness, the presence of interatrial defects/aneurysms or patent oval foramen should be taken into account.

Once in the left atrium, the most appropriate projection for advancing the guidewire into the left superior pulmonary vein is ${\mathrm{45}}^{\circ }$ with the probe rotated clockwise. A 50–${\mathrm{90}}^{\circ }$ projection is then indicated to allow the pigtail to reach the left atrial appendage. Correct placement should then be confirmed in multiple projections.

The size of the device should be reconfirmed at this point and it should be evaluated with a left atrial pressure of at least 12 mmHg for the risk of underestimating the size of the device.

Finally, prior to final device release, transesophageal ultrasound can be used to assess the presence of peri-device leaks and possible pericardial effusion at the end of the procedure.

### 3.3 Cardiac Computed Tomography

Multi-slice cardiac CT is a fundamental exam for procedural planning of percuntaneous or surgical LAAO and permits an accurate study of LAA and surrounding structures, also allowing for the selection of best device for the procedure.

**Fig. 2.**
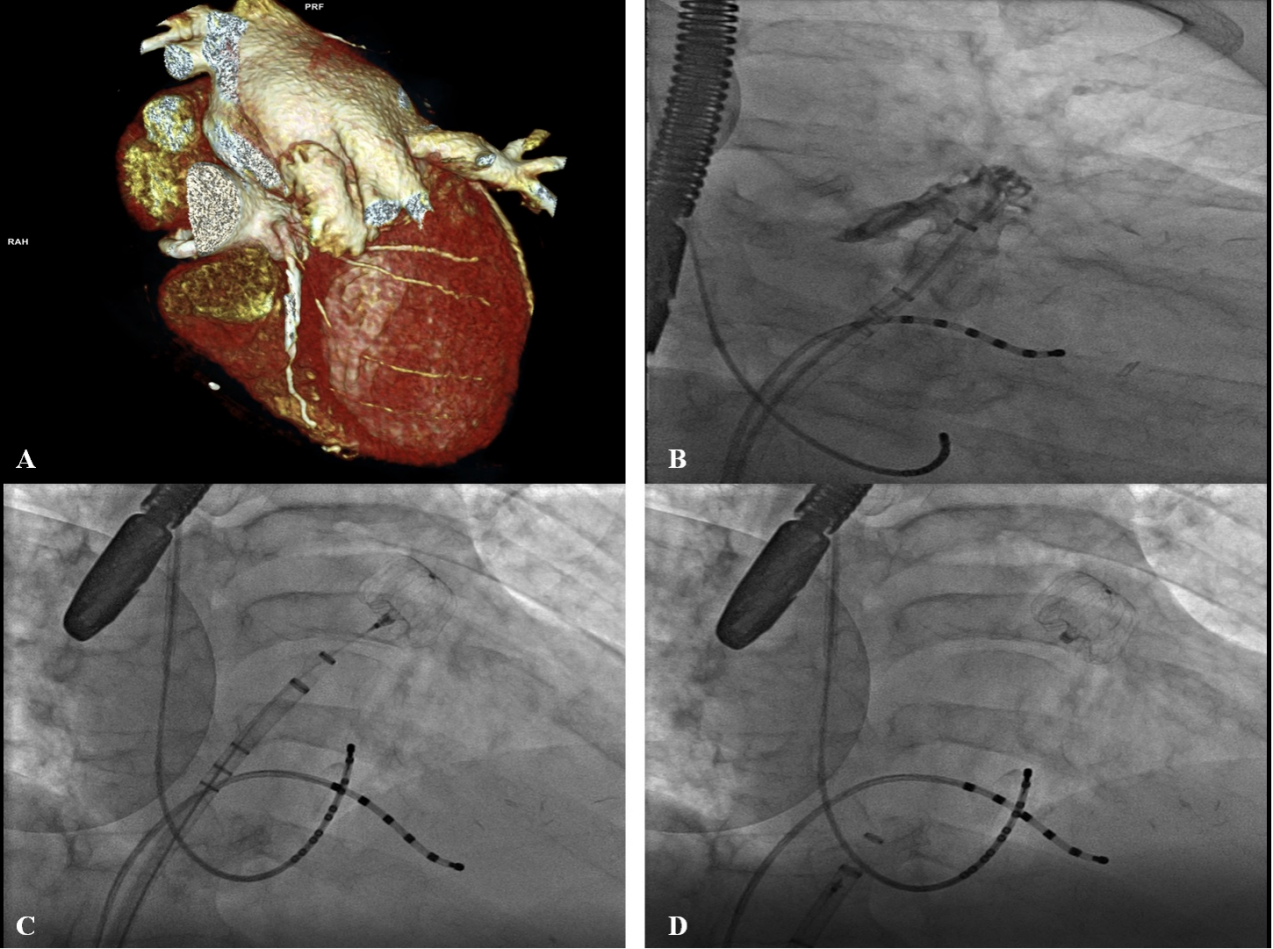
**Pre procedural imaging evaluation and fluoroscopic assessment of LAAO**. (A) Shows a pre procedural Cardiac CT assessment of LAAO. In this case it was necessary perform transseptal puncture quite low and anterior in the fossa ovalis for this specific anatomy (high an anterior large LAA with morphology of “reverse” chicken wing). (B–D) Describe during fluoroscopy the positioning of the device with double anterior curve sheath. Catheters in coronary sinus and “His” electrophysiological anatomical location were placed to guide the transseptal puncture during fluoroscopy and device placement in this particular case.

Cardiac CT has a high sensitivity (96%) and a high negative predictive value (100%) for the evaluation of thrombotic formations [17]. Once the cardiac CT images are obtained, post-processing is performed, which performs a multi-planar reconstruction of axial acquisitions. Finally, 3D reconstruction provides the ability to fully visualize the appendage and morphology. The multi-planar reconstruction also allows correct sizing of the device with different measurements that should be taken depend on the type of device selected and sometimes suggest information about different transeptal puncture position (Fig. 2) [18].

In particular Eng *et al*. [19] in a single center experience compared 3D CT to TEE before LAAO in 24 patients that were prospectively randomized. In patients undergone to 3D CT the LAAO procedures was more accurate in device selection accuracy, measurements and improves case planning.

### 3.4 Intracardiac Echocardiography 

Intracardiac echocardiography (ICE) is an alternative technique to TEE for percutaneous LAAO [20, 21].

Compared to TEE, it is more invasive and less sensitive in identifying thrombotic formations, but may be particularly useful in those patients contraindicated to TEE (esophageal diseases, such as stenosis or varices) or contraindications to deep sedation/intubation [20].

The ICE catheter, located at the level of the right atrium, allows visualization of most of the left atrium and LAA anatomy and size. It can be used to guide the transseptal puncture and to verify the occlusion, position and stability of the device implanted.

Currently the most commonly used ICE catheters are ViewFlex (Abbot Vascular, Santa Clara, CA, USA), which measures 9 French (Fr) and the AcuNav (Biosense Webter, Irvine, CA, USA) available in two different sizes (8 and 10 Fr).

Both, without the need for sedation, are introduced by peripheral venous access and positioned in the right atrium, initially in what is called the “Home View” and allowing visualization of the right structures. The left structures (aortic arch, long axis of the aortic valve, left ventricular outflow tract) are visualized by rotating the catheter clockwise. Continuing this rotation it is possible to visualize the fossa ovalis and by changing the depth it will provide an adequate projection for visualization of the interatrial septum and then for performing the transseptal puncture.

The presence of LAA thrombus should be explored and ruled out prior to the transseptal puncture. This can also be done by inserting the catheter into the coronary sinus or pulmonary artery. Of course, the left structures are best visualized by passing the ultrasound probe through the interatrial septum. The left atrial passage can be performed in the same transseptal puncture performed with the device guides or can help perform the transseptal puncture itself by positioning it in retroflexion. Once the transseptal puncture has been performed, the probe is positioned in front of the atrial appendage to allow the device to be inserted into the atrial appendage. The probe should then be moved to the superior left pulmonary vein or rotated inferiorly to assess different image angles [18].

## 4. Device Characteristics for Percutaneous Left Atrial Appendage Closure

The most common percutaneous LAAO devices available at the moment are: Watchman, Watchman FLX, Amplatzer Amulet, WaveCrest and Lambre.

The first device used for percutaneous LAAO (PLAATO - Percutaneous Left Atrial Appendage Occlusion) consists of a self-expanding cage with an occlusive membrane coated in its atrial surface by polytetrafluoroethylene. This device, while demonstrating clinical efficacy, had shown several drawbacks and was withdrawn from the market in 2006 [22].

WATCHMAN device (Boston Scientific, Natick, MA, USA) consists of a self-expanding titanium and nickel structure coated on the atrial surface by a polyethylene terephthalate (PET) membrane with fixation tines that anchor itself on LAA orifice. PET remains in contact with the blood in the atrial chamber and promotes healing and endothelialization. If re-positioning is required, the device may be partially or completely recaptured. Tree different sheaths are available: single curve, double curve, and anterior curve (the latter two provide greater assistance in device placement in LAA positioned more superiorly towards the aortic root).

This device exists in 5 different sizes (21, 24, 27, 31, 35) and is preassembled within a placement catheter (14 Fr). Two measures are essential to determine the correct device size: the “landing zone” measured from the circumflex coronary artery and the transition between smooth and trabecular atrial appendage, and the depth measured from the “landing zone” to the apex of the atrial appendage. 10%–20% oversizing is also indicated to ensure greater stability of the device.

The procedure is usually performed under general anesthesia and guided by fluoroscopy, TEE or ICE.

The new generation of Watchman devices is Watchman FLX. Compared to its predecessor, due to structural changes, it is less traumatic for LAA, easier to implant in superficial atrial appendages, more stable and with reduced risk of device-related thrombosis. It is available in 5 sizes (20, 24, 27, 31, 35 mm) [23].

The transseptal puncture (TSP) should be often performed in the posteroinferior segment of the fossa ovalis after adequate anticoagulation with heparin (ACT >250 seconds). However, the puncture may be individualized in certain anatomies. Similarly, not all centers use the approach to administer all heparin before TSP; in fact, in some centers 2500 units or half dose are administered upon venous access and remaining upon TSP.

Before the device is released, sizing should be confirmed by angiography and confirmed with TEE: correct placement (maximum device diameter must be at LAA ostium without excessively protruding into the left atrium), stability (Tug Test), correct size, proper occlusion (all lobes must be distal to the proximal portion of the device and no peri-device leaks should be visible after Color- Doppler assessment or contrast injection) (Fig. 2).

If the device is incorrectly positioned, if it is too distal it can be partially recaptured, if it is too proximal (or sizing is incorrect), it will need to be completely recaptured and the device replaced.

With the Watchman FLX device, it is also possible to correct a position that is too proximal with both partial and complete recapture.

The Amplatzer AMULET device (St. Jude Medical, St. Paul, MN, USA) consists of a self-expanding, sharp metal mesh that forms a distal lobe, which fits into LAA body and a proximal disc covering the ostium. The distal lobe has a diameter of 6 to 10 hooks designed to ensure anchorage stability, which is also aided by its radial strength and proximal disc traction. It exists in 8 different sizes (16, 18, 20, 22, 25, 28, 31, and 34 mm) and was developed based on the Amplazer Septal occluder (ASD) used in the closure of inter-atrial septum defects. The device is pre-loaded into a 12–14 Fr dual curve sheath. There are numerous studies in literature on clinical efficacy of this device [24, 25, 26, 27, 28, 29, 30, 31].

Device size selection must be based on two parameters: the “landing zone” that must be measured 1.2 cm distal to the ostial plane, and the depth that should be considered from the ostial plane to the bottom of LAA. It is also recommended to choose a size from 2 mm to 4 mm larger than the one calculated to improve the stability of the device.

Before device release it is essential to verify some points using TEE or fluoroscopy: appropriate placement (the lobe must be coaxial to the LAA and adjacent to the circumflex coronary artery), stability (Tug test), correct size, complete occlusion (disc must be adequately separated from lobe and peri-device flow to Color-Doppler or contrast injection should not be visualized).

If placement is unsatisfactory, both the disc and lobe can be retracted by re-positioning them. If during this maneuver it is necessary to withdraw them beyond the radiopaque markers of the device, the entire device will need to be removed.

The Wavecrest device (Coherex Medical Inc, Salt Lake City, UT, USA) consists of a foam-coated construction with a particular feature: radial force is not used for stability but 20 retractable hooks for fixation.

It is possible the use of 3 different sizes (22, 27, and 32 mm) and device is preassembled within a placement catheter (12 Fr).

It is possible to choose 4 different catheters shapes: single curve (${\mathrm{60}}^{\circ }$, ${\mathrm{75}}^{\circ }$ and ${\mathrm{90}}^{\circ }$) and anterior curve (${\mathrm{90}}^{\circ }$).

Device size selection must be based on two parameters: the “landing zone” that is measured from the circumflex coronary artery to the transition between the smooth and trabecular zones of LAA and LAA depth. It is recommended to select a size 3 to 8 mm larger than the calculated size in order to improve stability.

The procedure is generally conducted under general anesthesia under fluoroscopy and TEE or ICE.

Before device release, fluoroscopy or TEE testing should be performed: appropriate position (the lobe must be positioned in the proximal part of the atrial appendage), stability (with Tug Test), correct size, and proper exclusion (peri-device flow cannot be displayed during proximal contrast injection and no extravasation of contrast after distal injection).

If the device position is not satisfactory, the hooks can be retracted and the device can be completely removed and re-positioned.

Lambre (Lifetech Scientific [Shenzhen] Co., Ltd. Shenzhen, China) is composed of a proximal disc and a distal, nitinol lobe connected by a central structure that is the particular feature of this device, that allow a different angulation of the two structures without compromising the stability of the device.

There are two different device designs, indicated for different anatomies (single or dual lobe). Each type has different sizes (16,18,20,22,24,26,28,30, 32, 34, 36 for single lobe and 16, 18, 20, 22, 24, 26 for double lobe). The sheath has been manufactured in two forms: single curve and double curve.

For the single lobe device, measurements are based on: the “landing zone” measured by the circumflex coronary artery at the transition between the smooth and trabecular zones of the atrial appendage and depth must higher than 10 mm.

However, for the two-lobe device, measurement should be based on: lobe “landing zone” width approximately 1 cm distal to the atrial appendage bifurcation, bifurcation depth, lobe depth.

Over-sizing of 3 to 8 mm is recommended to improve device stability.

**Fig. 3.**
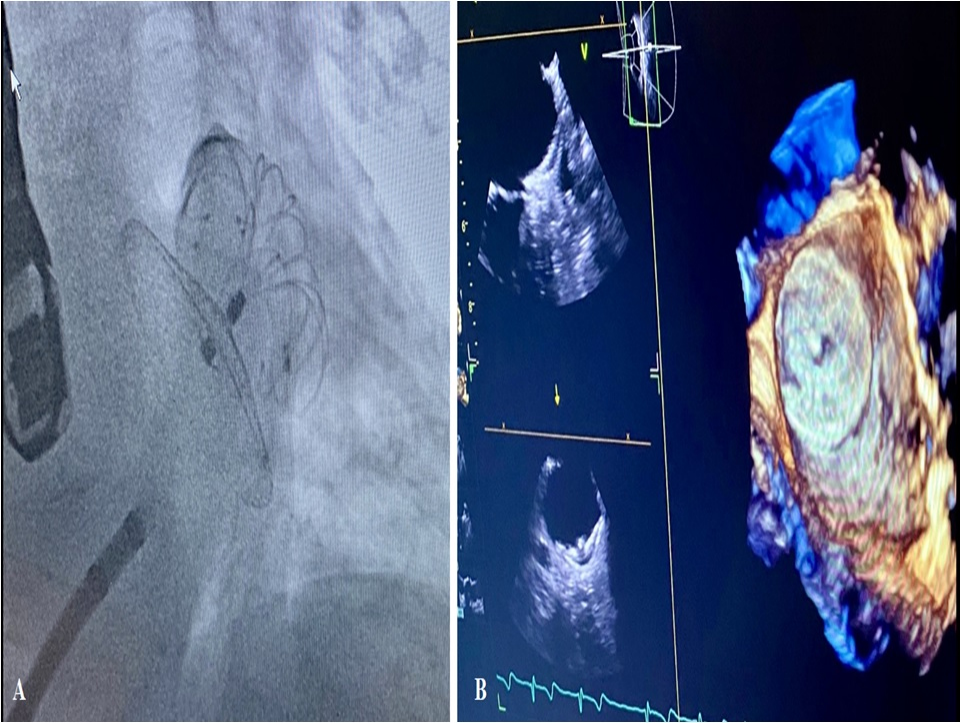
**TEE monitoring during percutaneous LAAO with Lambre device**. (A) Highlights device release. (B) Describes in 2 D and 3 D modality TEE the complete occlusion of LAA.

The procedure, typically performed under general anesthesia, should be performed under fluoroscopy and TEE or ICE. The measurement should be confirmed with angiography, and before releasing the device fluoroscopy or TEE should be used to evaluate correct positioning, stability (Tug test), correct size, and proper occlusion. If placement is not satisfactory, the device can be recaptured and repositioned [32] (Fig. 3).

## 5. Post Procedure Anti-Platelet Strategy

LAA post-closure anti-platelet regimens used starting from the ASAP Registry develop the same endothelialization concept as other “cardiac prostheses”, such as stents or devices used for oval foramen closure.

Studies of endothelialization of percutaneous LAA closure devices were performed on small canine specimens with different antithrombotic therapies and devices. Endothelization ranged between 28 and 90 days [33]. A similarity of device endothelization was shown between the canine and human specimens although significant variability was observed: compared to the healing process in animals, human healing seems to take longer and varies among patients [34].

Dual antiplatelet therapy with cardiospirin and clopidogrel/ticlopidine was tested in the ASAP Registry for 6 months post-implant in 150 Watchman device patients. The annual incidence of ischemic stroke was 1.7, while device-related thrombi occurred at a rate comparable to the percutaneous left atrial appendage group in protect-AF/PREVAIL studies, suggesting the effectiveness of dual antiplatelet therapy [10].

In the medical therapy arm of PROTECT-AF and PREVAIL studies, warfarin anticoagulation therapy was used for 45 days followed by cardioaspirin and clopidogrel for 6 months and then by single cardioaspirin. A comparable rate of stroke and systemic embolism was found between this group of patients and the group treated with Watchman device, while bleeding strokes were significantly lower in the Watchman group. These results lead to the safe use of warfarin post procedure in patients eligible for warfarin therapy [8, 9].

Following the ASAP Registry, dual anti-platelet therapy was recommended as a therapy scheme outside the U.S. thanks to the EVOLUTION trial: a prospective, multicenter study that collected data from clinical practice in the years 2013–2015 in 1025 Watchman patients [35]. At discharge, 27% of patients were treated with warfarin/NOAC, 60% with double antiplatelet therapy, 7% received single antiplatelet therapy, and 8% received no therapy. The annual ischemic stroke rate was 1.3%, while the device-related thrombosis rate was 2.8%. No association was found between these events and the assigned type of medical therapy. The WASP Registry investigating 201 patients between 2014 and 2015 showed comparable data [36].

In ACP Registry (2008–2013) 1047 patients undergoing percutaneous LAAO with AMPLATZER device. 16% of these patients were treated with double antiplatelet, 35% with single antiplatelet, 10% with anticoagulant (warfarin/NOAC) and antiplatelet, 17% with anticoagulation, 7% with low molecular weight heparin, 8% with no therapy. The annual rate of ischemic stroke and device thrombosis was 1.3% and 2.7%, respectively, which is comparable to other Watchman device studies [37].

In 2019 a study compared antiplatelet therapy versus anticoagulation therapy analyzing patients from PROTECT-AF, PREVAIL, CAP, ASAP, and EWOLUTION studies. No differences were observed in terms of major bleeding or thromboembolism between warfarin and antiplatelet therapy (91% double antiplatelet, 9% single antiplatelet). The only difference was in terms of device-related thrombosis: the antiplatelet group reported a higher and significant percentage of these events (3.1% vs 1.8%) even though thromboembolic complications were not reported. In the warfarin group 3 patients with device-related thrombosis developed thromboembolic complications [38].

In the EVOLUTION trial, 7% of patients were discharged with single antiplatelet therapy and 6% had no therapy. Ischemic stroke in these groups did not differ significantly from the rest of the treated groups. Conversely, in the RELEXAO study where percutaneous LAAO discharge therapy was single antiplatelet in 35.8% of cases and no therapy in 7.7% of cases, there was a higher device-related thrombosis rate and ischemic stroke (annual) compared to literature data (5.4% and 4% respectively) [39].

Korsholm K *et al*. [40] demonstrated in their experience 110 patients at high risk of bleeding who underwent percutaneous LAAO with the AMPLATZER Cardiac plug or Amulet were treated only with aspirin showed more reassuring data: 1.9% device-related thrombosis, 2.3% annual ischemic stroke rate and a lower incidence of major bleeding.

In addition the not insignificant resistance to clopidogrel should also be considered (the prevalence of clopidogrel in the literature ranges from 5% to 44%), which may have overestimated the need for double antiplatelet therapy [41].

Data about patient treated with NOAC after percutaneous LAAO are lacking. In the EWOLUTION trial 109 patients were treated with NOAC after percutaneous LAAO with no ischemic strokes recorded in this group [42]. In 2017 a retrospective multicenter study evaluated 214 patients who underwent percutaneous LAAO with Watchman device and were treated with NOAC after the procedure (46% apixaban, 46% rivaroxaban, 7% dabigatran and 1% edoxaban). Compared to a group of warfarin treated patients, the frequency of device-related thrombosis, post-procedural bleeding, or device-related thromboembolism and thrombosis was not significantly different in the two groups [42].

Recently Della Rocca *et al*. [43] demonstrated that after LAAO with Watchman device, half-dose of NOAC significantly reduced the risk of the thromboembolic and major bleeding events compared with a standard antiplatelet therapy in similar population of patients with high cardioembolic and bleeding risk.

Even if actually no consensus exists for the choice of the most safe and effective anticoagulant/antiplatelet strategy after percutaneous LAAO, Mazzone *et al*. [44] demonstrated regardless of antithrombotic therapy (dual antiplatelet therapy, OAT, single antiplatelet agent, a combination of antiplatelets and OAT or without any antithrombotic therapy) incidence of adverse events was low and the efficacy on embolic during the follow up was similar in all the patients treated with different drugs regimens.

## 6. Surgical Exclusion of the Left Atrial Appendage

A previously described, LAA is a common origin of cardiac-derived emboli and sometimes plays a role in AF triggering both [45, 46]. This dual role is the basis of the growing interest in research into methods for its surgical exclusion too. Surgical exclusion may be performed concurrently with sternotomy or minithoracotomy cardiac surgery or as a totally thoracoscopic (TT) isolated procedure through direct suture, amputation/suture, stapler and clip [47].

The first surgical LAA exclusion report for stroke prevention dates back to 1949 by Madden *et al*. [48] but only with the publication of Cox *et al*. [49] on the surgical treatment of AF using the COX-MAZE technique exclusion of LAA entered into surgical practice for its antiarrhythmic role.

The initial exclusion methods used in sternotomy, such as outer ligation, endocardial suture, amputation/suture, and stapler were unsatisfactory with for ligation and for amputation and suture as reported by Kanderian *et al*. [50].

Initial skepticism about the procedure also depended on reports that showed a stroke risk that was approximately 2-fold if the procedure was incomplete, compared to non complete exclusion [51].

Subsequent improvement in techniques and devices in addition to coding as a standard of success of the residual stump <1 cm to standardize results has helped to change the attitude of surgeons in favor of the combined process of exclusion.

Bakhtiary F *et al*. [52] demonstrated a new technique that allow a complete obliteration of the LAA in high-risk patients undergoing cardiac surgery the new technique.

A meta-analyses conducted in 2015 by Tsai *et al*. [53] in 3653 patients which showed lower mortality and incidence of stroke in patients who underwent concomitant LAA exclusion support the efficacy and safety of LAA exclusion in combined surgery.

In the LAAOS III study Whitlock *et al*. [54] 2021 demonstrated the most reliable exclusion methods in combined surgery: amputation and suture, double endocardial suture, stapler or clip resulted in a one-third reduction in the risk of stroke in patients subjected to heart surgery undergoing anticoagulation therapy.

In the field of minimally invasive mitral surgery, LAA exclusion has become common practice with suture [55] or with clips, as published by Alqaqa [56] with 98% satisfactory results in terms of success and effectiveness, as reported by Kurfirst *et al*. [57].

The recent advent of robotic surgery has presented a new challenge for the surgical exclusion of LAA, which has proved feasible with both double sutures with 87% success rates as reported by Ward *et al*. [58], and with clips with results that can be promising, as reported by Lewis *et al*. [59], recording success in 64 out of 68 patients (94%).

In the field of minimally invasive surgery, the last frontier today is the TT approach with the use of staplers or clips both as an isolated and combined procedure. The first description of TT LAA exclusion dates back to Blackshear *et al*. [60] with stapler and snare loop, the so-called LAPTONI procedure, but the first device available for the TT approach was the stapler in 1988, used by Di Sesa experimentally in 14 sheeps [61]. Incidence of complications and limitations have delayed its application in cardiac surgery up to the next generation staplers, which are more reliable and effective as reported by Ohtsuka with 93% success, 2 thoracotomy conversions and no stump in 63% of patients [62].

The first clip closure report dates back to 2008 with Fumoto’s experience in the animal model [63].

Actually the safest and most effective device for a thoracoscopic or minimally invasive approach is AtriClip device (Atricure, West Chester, Ohio) [64, 65]. In a series of 45 patients subjected to isolated TT LAA exclusion with AtriClip and non valvolar AF (mean follow-up of 16.9 months ± 9 months), there were neither procedural complications nor neurological events in the absence of antiplatelet/anticoagulation therapy during the follow up [66].

Indication to surgical exclusion can be achieved also in patients with very complex anatomy previous described by pre procedural assessment imaging (Cardiac CT, TEE) difficult to occlude with a classical percutaneous approach [67].

Furthermore, the surgical exclusion of LAA in patients with absolute contraindication to anticoagulant and antiplatelet therapy, even in the short term, represents a valid therapeutic alternative to minimize the procedural risk and optimize the outcome [68].

**Fig. 4.**
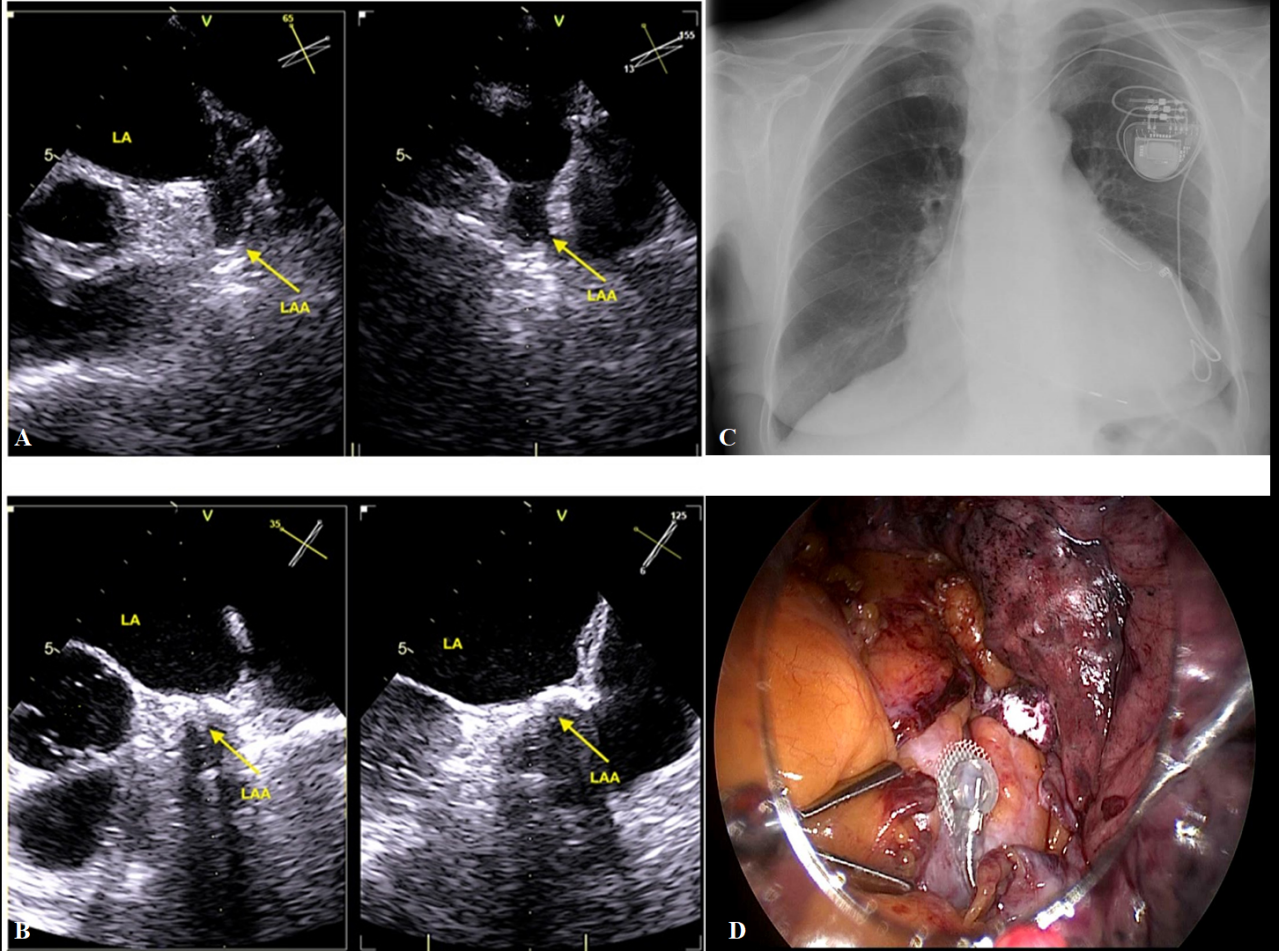
**A case of TT concomitant LAA exclusion closure and left ventricular epicardial lead implantation**. (A,B) Describes TEE monitoring before and after TT LAA exclusion with AtriClip device. (C) Describes antero-posterior chest x-ray image after LAA exclusion and epicardial LV implantation. (D) Highlights image of LAA exclusion and epicardial left ventricular epicardial lead before left lung was re-expansion.

This procedure can be performed successful concomitant procedure as TT epicardial left ventricular lead implantation for cardiac pacemaker/implantable automatic defibrillator cardiac resynchronization device also [69] (Fig. 4).

Surgical exclusion of LAA can allow electrical isolation of the tissue with the aim to treat complex atrial arrhythmias non responsive to percutaneous ablative procedure [70, 71].

The combination of LAA exclusion and contextual electrical isolation is useful in hybrid ablation procedures for the treatment of persistent AF as proposed by Richardson *et al*. [72]. In the context of bilateral ablation of TT AF ablation (TT MAZE), as reported by Van Laar *et al*. [73] in a multicenter study, a clip device was successfully implanted in 95% of cases out of 222 patients without device complications and freedom from neurological events in 99.1%. Encouraging results have also been reported in the monolateral hybrid ablation approach of AF ablation and LAA exclusion [74]. Probably the ideal device and technique for LAA exclusion are not yet available and research in this field is rapidly evolving.

## 7. Hormonal Role of LAA and LAA Exclusion

The endocardial occluders are aimed to create a mechanical barrier between the LAA and LA without fully eliminating the LAA body; conversely, the epicardial excluders cause necrosis and fibrosis of the LAA body distal to the point of ligation or clipping. Hence, some studies investigated the hormonal implications of epicardial LAA ligation resulting in temporary fluid retention and long term blood pressure reduction in patients with AF [75].

The LAA is richly innervated by both parasympathetic and sympathetic fibers. Both atrial appendages participate in reflex responses to stretch, although removal of the right or both appendages seems to have a greater impact than LAA removal alone. Therefore, epicardial closure techniques leads to progressive atrophy and fibrosis of the appendage with subsequent loss of neural and hormonal element.

In fact, epicardial closure causes decrease of catecholamines, angiotensin II and aldosterone starting from 24 hours till 3 months after the procedure, with following blood pressure decrease. On the contrary, natriuretic peptides, renin, insulin and adiponectin increase [76].

However, after epicardial closure, it is possible to deal with to two pictures: acute changes in natriuretic peptides may mediate short-term alterations in blood volume and serum sodium in the periprocedural period, but these hormone levels may return to baseline values within a few months indicating that any short-term neurohormonal effects of LAAO are mediated by natriuretic peptide pathway this mechanism. This finding is not surprising, given that the cardiac sources of both peptides are widely distributed in the atria and elsewhere, and may compensate for the loss of the LAA contribution. Another pathway, which may account for more long-term effects, may be due to the interruption or modification of neural reflexes, either by destruction of afferent fibers within the LAA or by injury to peri-LAA ganglionated plexi during extrernal ligation [77].

These findings suggest to carefully evaluate the profile of every patient to submit to LAAO, since blood pressure reduction, if not limited to first post procedural period, may impact of patients with heart failure, while could be useful in hypertensive patients. Moreover, some post-procedural therapeutic modifications may be necessary, such as antihypertensive and hypoglycemic drugs dose reduction and introduction of diuretics for fluid retention.

## 8. Conclusions

Percutaneous LAAO and more recently TT surgical LAA exclusion demonstrated a significant decrease in major and minor bleeding in patients with contraindications to long-term OAT while maintaining an efficacy in the prevention of cardioembolism.

A multidisciplinary approach with a careful evaluation of pre-procedural imaging (cardiac CT, TTE TEE) and intraprocedural (TEE, ICE) imaging/monitoring is required to successfully plan the correct procedure (percutaneous LAAO or TT surgical LAA exclusion) for the patient’s anatomy.

Post procedure antiplatelet/OAT strategy remains a point of interest for further studies with long-term follow up. A particular care should be taken in patients with heart failure, especially in case of epicardial closure, because of post-procedural hormonal changes with fluid retention and blood pressure reduction.
